# Editorial: Novel and potential biomarkers for prediction of outcome in patients with chronic and acute coronary heart disease, volume II

**DOI:** 10.3389/fcvm.2024.1432580

**Published:** 2024-05-28

**Authors:** Dennis W. T. Nilsen, Frederic Kontny, Hugo ten Cate

**Affiliations:** ^1^ Department of Cardiology, Stavanger University Hospital, Stavanger, Norway; ^2^ Department of Clinical Science, University of Bergen, Bergen, Norway; ^3^ Consultant Cardiology, Drammen Heart Center/Stavanger University Hospital, Drammen, Norway; ^4^ Department of Internal Medicine and Biochemistry and Thrombosis Expertise Center, Maastricht University Medical Center and CARIM School for Cardiovascular Diseases, Maastricht, Netherlands; ^5^ Center for Thrombosis and Hemostasis, Gutenberg University Medical Center, Mainz, Germany

**Keywords:** biomarker, thrombosis, cardiovascular disease, coronary artery disease, inflammation

Editorial on the Research Topic Novel and potential biomarkers for prediction of outcome in patients with chronic and acute coronary heart disease, volume II

Upon completion of the thematic series on “Novel and Potential Markers for Prediction of Outcome in Patients with Chronic and Acute Coronary Heart Disease”, comprising a total of 14 articles, the editors try to make up the balance.

The idea about this series stems from diagnostic uncertainties of the medical specialist: are there biomarker tools that can help to better stratify risks, to further optimize individual patient management? Like in many patients with a given chronic, or acute on chronic vascular disease, marked heterogeneity is a hallmark that poses many management dilemmas. While most elements in the management pathway, including recommended pharmacotherapy, have been protocolized according to international guidelines, we know from experience that many individual patients do not perfectly fit these guidelines. In addition, patients may also want to deviate from recommended treatments, often for good reasons (e.g., concerns about side effects, costs).

Patient management remains an individually tailored art, when time allows. Shared decision making is part of modern patient management, again for very good reasons. Many patients are well informed about indications, contra-indications and side effects of therapies that we propose. Thus, for physicians and patients it is becoming more important to have available tools to support management decisions.

Blood biomarkers have the potential to support our decision making but in practice, relatively few have made it to routine clinical application. In the series of papers accumulated over the past 7 years, none of the investigated biomarkers have made it to the clinic, at least not for the indication that was addressed. For example, d-dimer, a split product from crosslinked fibrin and a biomarker for ongoing blood coagulation and fibrinolysis *in vivo*, is an established marker in the initial evaluation of patients with suspected venous thromboembolism (VTE). In conjunction with a low *a priori* risk assessment, a negative d-dimer level helps to rule out VTE. In the paper by Jiang et al., d-dimer was studied in the setting of cardiogenic shock after acute myocardial infarction (AMI). In their study an elevated d-dimer level added power to traditional risk scores in that setting. Other studies have shown that elevated d-dimer levels are also predictive of mortality in the context of peripheral artery disease (PAD) patients (1). Nevertheless, d-dimer levels have not yet been incorporated in guidelines for cardiovascular disease (CVD) management, although validated commercial assays are widely available already for a long time (2).

Another promising biomarker, GDF-15, was independently associated with coronary plaque (volume) in a small study assessing different blood biomarkers in relation to CVD, confirming its potential impact in CVD risk prediction Royston et al. This biomarker appears to be at the threshold of clinical implementation, supported by its predictive power in the setting of AF, anticoagulation related bleeding and even death (3). Nevertheless, GDF-15 is not routinely used in the management of patients with coronary artery disease (CAD) or other vascular disorders.

More conventional biomarkers like elevated glucose levels, either in the context of impaired glucose tolerance or diabetes mellitus type 2 Schmitz et al. or as element of stress hyperglycemia Alkatiri et al. have been repeatedly linked to poor outcome. In general, the marker “glucose” has been part of routine blood testing, at least in those known with diabetes and while there is little discussion about the clinical relevance of perturbed glucose regulation, glucose levels are not routinely used for *predictive* purposes in patients with CVD.

The good thing about glucose is that it is readily available worldwide. The same holds true for biomarkers like potassium Ke et al. (low) hemoglobin or (high) leukocyte count, or even for neutrophil/lymphocyte ratio and monocyte count (4), Chi et al. that can be derived from automated analytic methods, also quite widely available. Ratios of inflammatory cells in conjunction with lower hemoglobin point to chronic inflammation as an underlying mechanism that alters bone marrow production. In conjunction with an elevated C-reactive protein (CRP) level, such biomarker patterns may become useful in dissecting low from higher risk of future CV events in our patients. Again: potentially!

In practice, many of our colleagues, we included, will ignore the longer-term impact of the mentioned biomarkers; some biomarkers, including glucose and potassium, require immediate attention, but are ignored for their predictive potential. Cell counts will typically be interpreted in the context of that moment: is there evidence of inflammation due to infection? Or vasculitis? Or as element of inflammation during myocardial infarction or acute VTE? In the chronic setting, maybe CRP will be measured on occasion, but mostly with the idea to rule out concurrent infections, rather than with the intention to guide start of colchicine or statins.

Same for d-dimers: while elevated d-dimer levels predict thrombotic CV events in patients with PAD, this biomarker is not yet used as a tool to start anticoagulant treatment with dual pathway inhibition (aspirin plus low dose rivaroxaban), while elevated clotting may provide a good proxy for thrombosis risk and a tool for assuming focused therapy.

The excuse usually is that “promising” blood biomarkers still need to be converted into practical and better standardized methods, to be validated in clinical trials with solid endpoints. This is one of the reasons that another biomarker that has been promising for decades, thrombin generation analysis, is still largely absent from the routine clinical laboratory (5). Delayed clinical translation may of course also be due to the laborious nature of a test, and automation or simplification as point of care test, is always helpful to promote diagnostic implementation.

Another excuse is that we need to keep searching for the holy diagnostic grail and we can argue that we should await further exploration of modern “omics” techniques: indeed, proteomics and transcriptomics have yielded more promising biomarkers in CVD [e.g., Liu et al.]. The main advantage is that these unbiased shotgun approaches may help to identify specific patterns among patients (through identification of endotypes) that could reduce the degree of individual patient heterogeneity and eventually improve tailored management. One can also anticipate that panels of biomarkers have added diagnostic value.

One way forward may be to better align diagnostic and pharmaceutical industry with clinical and translational investigators at an early stage of diagnostics or drug development. A reasonably successful example of such alignment is the application of high sensitivity CRP as marker to risk stratify patients with CVD for application of anti-inflammatory therapy (6). An example where the use of biomarkers like d-dimer could have been helpful is in the development of dual pathway inhibition (DPI) in patients with severe CAD or peripheral artery disease. In the latter setting, physicians still struggle with the decision-making process regarding residual risk. Which patients require DPI and in whom is continuation of a single or combined antiplatelet therapy sufficient or optimal (7)? Biomarkers for clotting, e.g., d-dimer, or activated platelets, e.g., soluble P-selectin, may be relevant for risk stratification, but studies that support such biomarker use are absent. Such studies need to be done!

## References

[1] Kremers B Wübbeke L Mees B Ten Cate H Spronk H Ten Cate-Hoek A. Plasma biomarkers to predict cardiovascular outcome in patients with peripheral artery disease: a systematic review and meta-analysis. Arterioscler Thromb Vasc Biol. (2020) 40(9):2018–32. 10.1161/ATVBAHA.120.314774 32640905 PMC7447177

[2] Wauthier L Favresse J Hardy M Douxfils J Le Gal G Roy PM D-dimer testing: a narrative review. Adv Clin Chem. (2023) 114:151–223. 10.1016/bs.acc.2023.02.006 37268332

[3] Shoemaker MB Stevenson WG. The ABC death risk score: is it time to start measuring GDF-15? Eur Heart J. (2018) 39(6):486–7. 10.1093/eurheartj/ehx642 29136146

[4] Meeuwsen JAL Wesseling M Hoefer IE de Jager SCA. Prognostic value of circulating inflammatory cells in patients with stable and acute coronary artery disease. Front Cardiovasc Med. (2017) 4:44. 10.3389/fcvm.2017.00044 28770211 PMC5509763

[5] Binder NB Depasse F Mueller J Wissel T Schwers S Germer M Clinical use of thrombin generation assays. J Thromb Haemost. (2021) 19(12):2918–29. 10.1111/jth.15538 34592058 PMC9292855

[6] Ridker PM Rane M. Interleukin-6 signaling and anti-interleukin-6 therapeutics in cardiovascular disease. Circ Res. (2021) 128(11):1728–46. 10.1161/CIRCRESAHA.121.319077 33998272

[7] Galli M Franchi F Rollini F Ortega-Paz L D'Amario D De Caterina R. Dual pathway inhibition in patients with atherosclerotic disease: pharmacodynamic considerations and clinical implications. Expert Rev Clin Pharmacol. (2023) 16(1):27–38. 10.1080/17512433.2023.2154651 36455906

